# Longitudinal adaptations in neural and behavioral systems following hearing restoration using cochlear implants

**DOI:** 10.1073/pnas.2613077123

**Published:** 2026-09-28

**Authors:** Shimin Mo, Claude Alain, Andrew Dimitrijevic

**Affiliations:** ^a^ Institute of Medical Science, Temerty Faculty of Medicine, University of Toronto, Toronto, ON M5S 1A8, Canada; ^b^ Evaluative Clinical Sciences Platform, Sunnybrook Research Institute, Toronto, ON M4N 3M5, Canada; ^c^ Rotman Research Institute, Baycrest Academy for Research and Education, Toronto, ON M6A 2E1, Canada; ^d^ Department of Psychology, University of Toronto, Toronto, ON M5S 3G3, Canada; ^e^ Music and Health Research Collaboratory, Faculty of Music, University of Toronto, Toronto, ON M5S 2C5, Canada; ^f^ Department of Otolaryngology-Head and Neck Surgery, Temerty Faculty of Medicine, University of Toronto, Toronto, ON M5S 1A1, Canada; ^g^Communication Sciences and Disorders, College of Liberal Arts and Sciences, Wayne State Universsity, Detroit, MI 428201; ^h^ Biological Sciences, College of Liberal Arts and Sciences, Wayne State University, Detroit, MI 428201

**Keywords:** cochlear implant, longitudinal study, speech neural tracking, selective attention, ecological validity

## Abstract

Cochlear implants (CIs) restore hearing in people with severe-to-profound hearing loss, but how behavioral and neural adaptations unfold after implantation remains unclear, particularly during continuous speech processing. Most longitudinal studies of postimplant plasticity have used simple auditory stimuli, limiting insight into continuous-speech processing in complex environments. We followed 19 newly implanted adult CI users at activation and at 3, 6, and 12 mo after activation. At each session, participants completed speech recognition testing in quiet and noise, a spatial triple-digit test, hearing-related quality-of-life measures, and electroencephalography during an ecologically valid continuous-speech selective-attention task with competing distractors. Neural analyses focused on temporal response functions to task-relevant and task-irrelevant speech envelopes and changes in scalp field topography. Speech recognition improved over time, and spatial-separation benefits emerged by 6 mo. Neural tracking of task-relevant speech was stronger and emerged earlier than tracking of task-irrelevant speech, and scalp topographies changed significantly across sessions. Stronger task-relevant neural tracking was associated with better speech recognition, better spatial listening, and higher quality of life. The largest behavioral and neural gains occurred early after activation and plateaued by approximately 6 mo. These findings provide a functionally relevant account of postimplant adaptation and establish longitudinal continuous-speech neural tracking as a useful framework for studying rehabilitation after sensory restoration.

## Significance

Successful cochlear implant (CI) use requires more than hearing sounds; it also requires learning to follow speech in busy everyday settings. However, much of what we know about brain adaptation after implantation comes from studies using simple tones, words, or sentences, which capture only part of everyday listening. In this study, newly implanted CI users listened to natural stories while competing speech played in the background. We found that cortical tracking of natural speech strengthened most during the first months after implantation, largely stabilized by about 6 mo, and was related to speech perception, spatial listening, and hearing-related quality of life. These findings show how naturalistic neuroscience approaches can reveal functionally relevant aspects of auditory adaptation after cochlear implantation.

A cochlear implant (CI) is a neural prosthesis that bypasses damaged inner ear hair cells and directly stimulates the auditory nerve. It is widely regarded as one of the most successful neural prostheses, with more than one million individuals with severe-to-profound hearing loss having received a CI worldwide to date (1). Substantial benefits are reported in both objective speech perception performance and subjective self-reported hearing outcomes (2–5). However, these outcome domains are often only weakly aligned: Standard clinical speech perception tests frequently show weak correlations with patient-reported quality of life (6), suggesting that commonly used measures capture only a subset of the processes that support successful real-world communication. This mismatch highlights a more fundamental gap in our understanding of how the brain supports everyday listening after cochlear implantation.

Everyday listening requires continuously transforming complex, dynamic acoustic input into meaningful percepts amid competing sounds and attentional demands—processes that are only partially captured by brief, highly controlled clinical tests (6). This limitation spurred growing interests in objective measures that better reflect speech processing under real-world listening conditions. Among objective measures, the temporal response function (TRF) is particularly promising because it quantifies how the brain tracks continuous speech over time. Conceptually, a TRF estimates the relationship between changes in the speech signal, such as fluctuations in the acoustic envelope, and the listener’s electroencephalogram (EEG) response at different time delays. A stronger TRF indicates that ongoing neural activity is more reliably synchronized with the speech stream, whereas the timing and spatial distribution of the TRF provide information about the neural processes supporting speech encoding. Because TRFs can be measured while listeners listen to naturalistic continuous speech, they offer an objective index of speech processing under conditions that more closely approximate everyday listening than brief, isolated words or sentences in clinical speech tests. Previous studies have shown that TRFs can serve as objective indices of speech intelligibility in quiet and noisy listening conditions (7–15), selective attention (16, 17), and listening demand (18). TRF measures have also been reported to correlate with cortical auditory evoked potentials (19).

Objective measures using TRFs for realistic speech are especially important in cochlear implantation, where benefit does not emerge uniformly across individuals or outcome domains. Despite the effectiveness of cochlear implantation in restoring auditory perception, postimplantation outcomes remain highly variable among CI users (20–24). Speech recognition benefit with a CI requires the brain to learn to interpret a spectrally degraded and temporally altered signal, a process likely involving experience-dependent plasticity across auditory and higher-order cortical systems over time (25–27).

Longitudinal designs are essential for characterizing neural plastic trajectories (28) and determining when meaningful behavioral and neural changes occur. However, relatively few studies have tracked CI adaptation over time. Behaviorally, a meta-analysis of 22 studies reported a nonlinear trajectory for speech recognition outcomes, including word recognition in quiet, and sentence recognition in quiet and in noise, across the first 24 mo after implantation (4). Across these measures, performance improved most rapidly within the first 3 mo and then tended to plateau. A similar pattern has been shown for quality-of-life measures (29).

In contrast, electrophysiological studies have provided a less consistent account of postimplant cortical adaptation. Only a small number of studies have used electrophysiological measures to characterize the trajectory of auditory cortical change following CI implantation (21, 30–32). Sandmann et al. (32) reported a rapid improvement in sensory-evoked responses to frequency-modulated tones at about 100 ms (i.e., the N1 wave, 100 ms poststimulus onset) after 8 wk of CI use, with no further improvement thereafter. By comparison, Purdy and Kelly (21) did not observe a clear group-level trajectory in N1 amplitude over time using tone bursts at three different frequencies and instead reported substantial interindividual variability. Other studies using simple tones showed rapid N1 enhancement after implantation, but their sample sizes were small (N = 1 or 2) (30, 31). Taken together, these findings suggest that neural adaptation may occur rapidly when assessed with simple tonal stimuli, but they do not readily account for the more gradual improvements observed in behavioral speech perception.

One possible explanation for this discrepancy is that prior electrophysiological studies relied on highly simplified stimuli that do not engage the processes required for continuous speech perception, such as auditory object formation, sustained attention, and segregation of competing sound sources (33–35). As a result, existing neural measures may primarily reflect early sensory encoding rather than the higher-order processes that support speech perception in everyday environments. This raises the possibility that the time course of neural adaptation may differ when assessed under more ecologically valid listening conditions.

The present study used TRFs to quantify how CI users process continuous speech and how this processing changes over time, from CI activation to 1 y postimplantation. By combining a longitudinal design with a continuous-speech paradigm, this approach allows us to track experience-dependent changes in the neural encoding of behaviorally relevant auditory input under conditions that more closely approximate everyday listening. The integration of continuous speech and TRFs offers two key advantages. First, continuous speech more closely approximates everyday listening, in which CI users must process unfolding, cohesive phrases rather than meaningless isolated words. Second, continuous-speech paradigms are particularly well-suited for longitudinal studies because different story passages can be presented across sessions while preserving the same overall task demands, thereby reducing the likelihood that observed improvement is driven primarily by repeated exposure or stimulus-specific learning. In the present study, we asked two questions: 1) what the time course of neural and behavioral adaptation to CI use is when assessed with continuous, attentionally demanding speech, and 2) to what extent do neural indices derived from this paradigm relate to conventional clinical measures and patient-reported outcomes. By repeatedly measuring behavioral and neurophysiological responses within the same individuals, this study aimed to characterize the time course of postimplant adaptation at both the behavioral and neural levels and to determine whether these measures relate to conventional clinical and patient-reported outcomes.

## Results

**Fig. 1.**
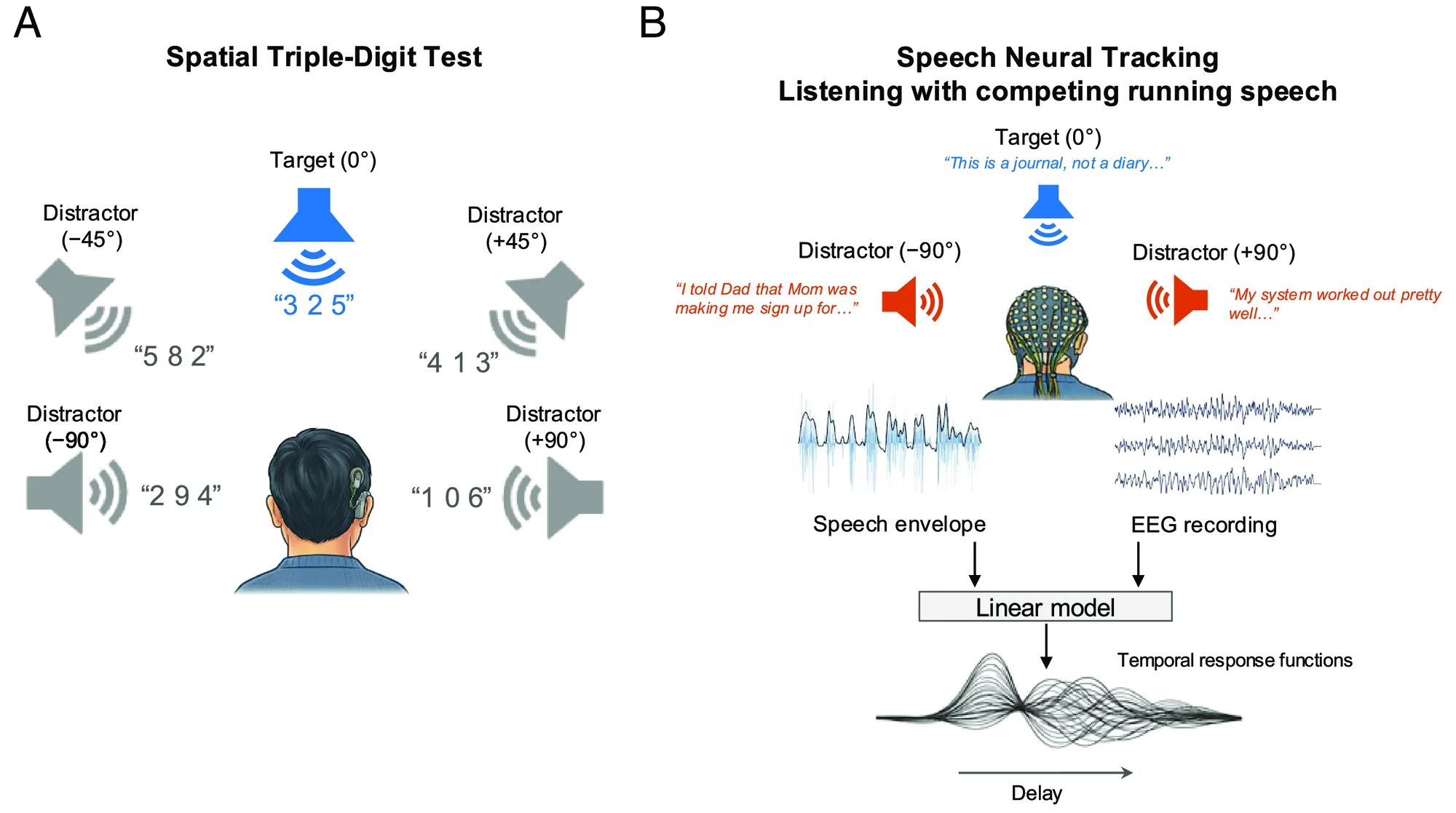
Experimental design. (*A*) STDT. Participants listened to and reported a series of triple-digit numbers played from the target speaker at 0° azimuth. They also heard distractor triple digits at varied spatial locations (±45° and ±90° azimuth) with the same or different voice identity (same-voice distractor, SV: distractor speaker identical to the target speaker; different voice distractor, DV: distractor voice created by shifting the target voice downward by three semitones). Participants completed four conditions, each representing a unique combination of distractor voice and spatial location: DV45, DV90, SV45, and SV90. Distractor intensity was adjusted adaptively to estimate the signal-to-noise ratio (SNR) corresponding to 50% accuracy. (*B*) Naturalistic listening paradigm. Participants were asked to attend to an audiobook presented from the target speaker under three conditions: Quiet (target speech only), DV90, and SV90. In the DV90 and SV90 conditions, participants were also instructed to ignore the distractors. Each condition lasted approximately 15 min. Manipulations of distractor locations and voice were identical to STDT, except for the stimulus content. Each distractor stream contained a story segment from a different audiobook series, and distractor materials were never used as targets.

We addressed three questions. First, how do behavioral measures of hearing function, including hearing-related quality of life (Cochlear Implant Quality of Life survey [CIQOL]), speech perception (AzBio sentence test), and spatial listening (Spatial Triple-Digit Test [STDT]; Fig. 1*A*), improve over the first year after CI activation? Second, how do neural responses to continuous speech (Fig. 1*B*) show corresponding longitudinal changes, particularly for task-relevant relative to task-irrelevant input? Third, are these neural measures associated with conventional clinical outcomes and patient-reported benefit?

### Behavioral Measures.

#### CI-related quality-of-life benefits emerged early and plateaued by 6 mo.

**Fig. 2.**
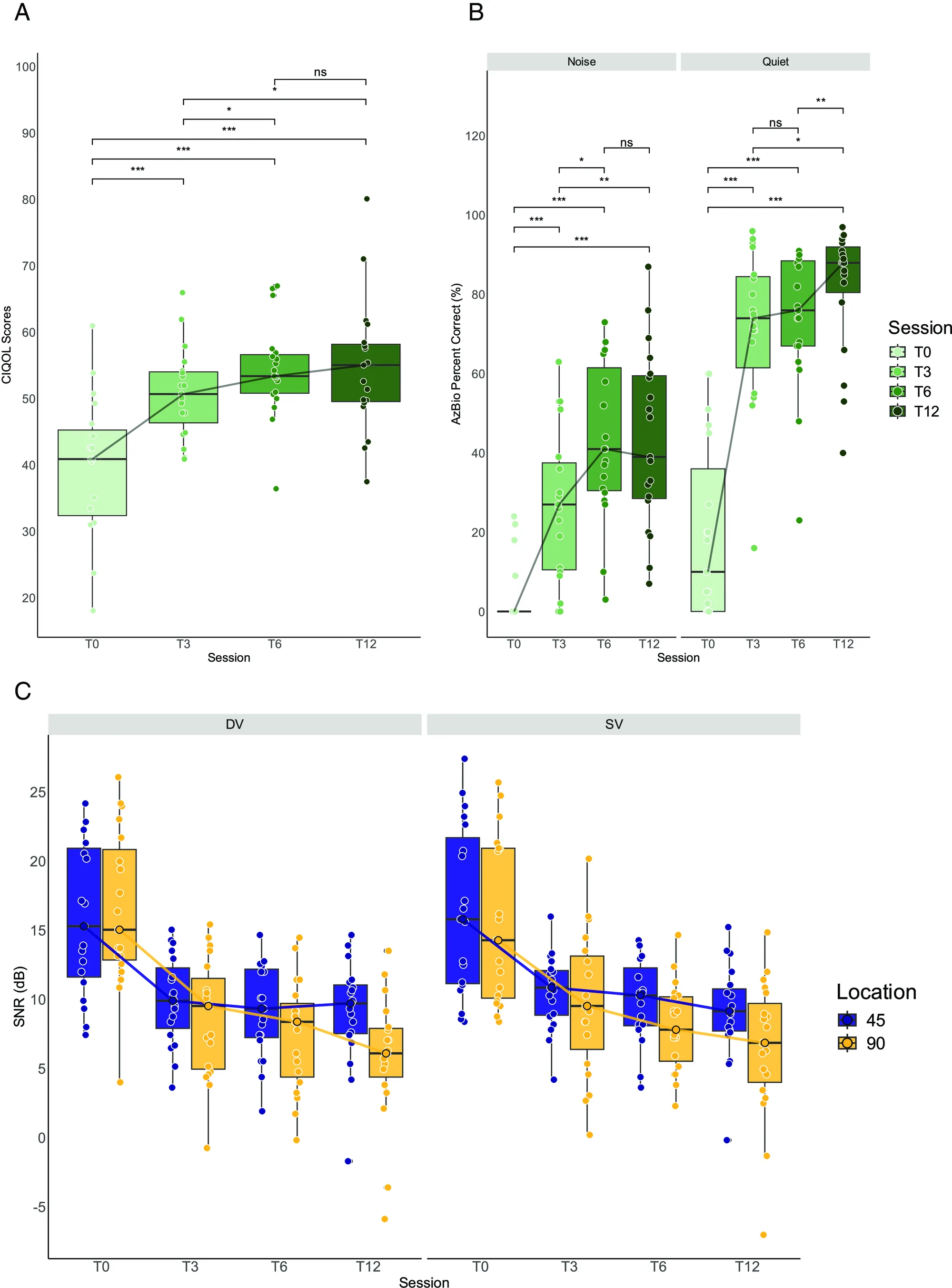
Longitudinal changes in behavioral outcomes after cochlear implant activation. (*A*) CIQOL composite score across sessions (T0, T3, T6, T12). (*B*) AzBio speech perception performance (percent correct) across sessions in Noise and Quiet conditions. (*C*) STDT speech reception thresholds (SNR, dB) across sessions for DV and SV listening conditions at 45° and 90° spatial separations. Lower SNR indicates better performance. In all panels, each dot represents an individual participant. Box-and-whisker plots show the median (horizontal line), interquartile range (box; 25th to 75th percentiles), and whiskers extending to the most extreme values within 1.5× the interquartile range. Overlaid lines connect the median across sessions to illustrate longitudinal change. Horizontal brackets indicate pairwise comparisons; ns = not statistically significant, *P < 0.05, **P < 0.005, ***P < 0.001.

To assess whether participants experienced functional benefits after cochlear implantation, we examined longitudinal changes in CI-related quality of life over the first year of device use. CIQOL composite scores increased significantly across sessions (Fig. 2*A*), *F*(3, 54) = 18.63, *P* < 0.001, $\eta_{G}^{2}=0.333$, with the largest improvement occurring between T0 (at activation) and T3 (3 mo after activation), continued but smaller gains from T3 to T6 (6 mo after activation), and no significant change from T6 to T12 (12 mo after activation). False discovery rate (FDR) corrected comparisons confirmed significantly lower scores at T0 than at all later sessions, all *P* < 0.001, and a further increase from T3 to T6, *P* = 0.017. These findings indicate that perceived CI-related quality-of-life benefits emerged early after implantation and plateaued by approximately 6 mo.

#### Speech recognition improved rapidly after activation, with condition-specific recovery trajectories.

To evaluate behavioral improvement in speech understanding, we examined changes in AzBio sentence recognition from activation to 12 mo postimplantation. AzBio performance was significantly higher in quiet than in noise, *F*(1, 18) = 187.6, *P* < 0.001, $\eta_{G}^{2}=0.45$, and improved across sessions, *F*(3, 54) = 77.13, *P* < 0.001, $\eta_{G}^{2}=0.55$ (Fig. 2*B*). This improvement was not uniform across listening conditions, as reflected by a significant Condition × Session interaction, *F*(3, 54) = 11.76, *P* < 0.001, $\eta_{G}^{2}=0.083$. In the Quiet condition, sentence recognition increased sharply from T0 to T3 and continued to improve by T12, with T12 scores significantly higher than both T3 and T6. In the Noise condition, performance also improved markedly after activation, with additional gains from T3 to T6, but then stabilized between T6 and T12. Overall, AzBio performance showed substantial postimplant improvement in both listening conditions, with a more extended trajectory in quiet and a plateau by 6 mo for speech-in-noise.

#### Spatial speech-understanding thresholds continued to improve across the first year.

To assess adaptation in spatial hearing under competing-speech conditions, we examined whether participants improved at identifying target digits at lower SNR during the first year of CI use. STDT thresholds improved significantly across sessions, *F*(3, 54) = 36.48, *P* < 0.001, $\eta_{G}^{2}=0.367$, indicating that participants required progressively less favorable (i.e., lower) SNRs to perform the task over time (Fig. 2*C*). Thresholds were also lower when distractors were located at 90° than at 45° azimuth, *F*(1, 18) = 12.11, *P* = 0.003, $\eta_{G}^{2}=0.029$, whereas the effect of distractor identity was not significant, *F*(1, 18) = 3.66, *P* = 0.071. Importantly, the spatial separation effect became more pronounced with CI experience, as reflected by a significant distractor location × session interaction, *F*(3, 54) = 4.10, *P* = 0.021, $\eta_{G}^{2}=0.014$. Follow-up comparisons showed that the 45° to 90° difference was not reliable at T0 or T3, but became significant at T6 and T12, suggesting that the benefit of spatial separation emerged gradually. Unlike CIQOL and AzBio, which showed rapid early improvements followed by later stabilization, STDT thresholds continued to improve across the first year, indicating a more prolonged adaptation trajectory for spatial listening under competing-speech conditions.

### Speech Neural Tracking.

#### Target-speech neural tracking strengthened over the first 6 mo after cochlear implantation.

**Fig. 3.**
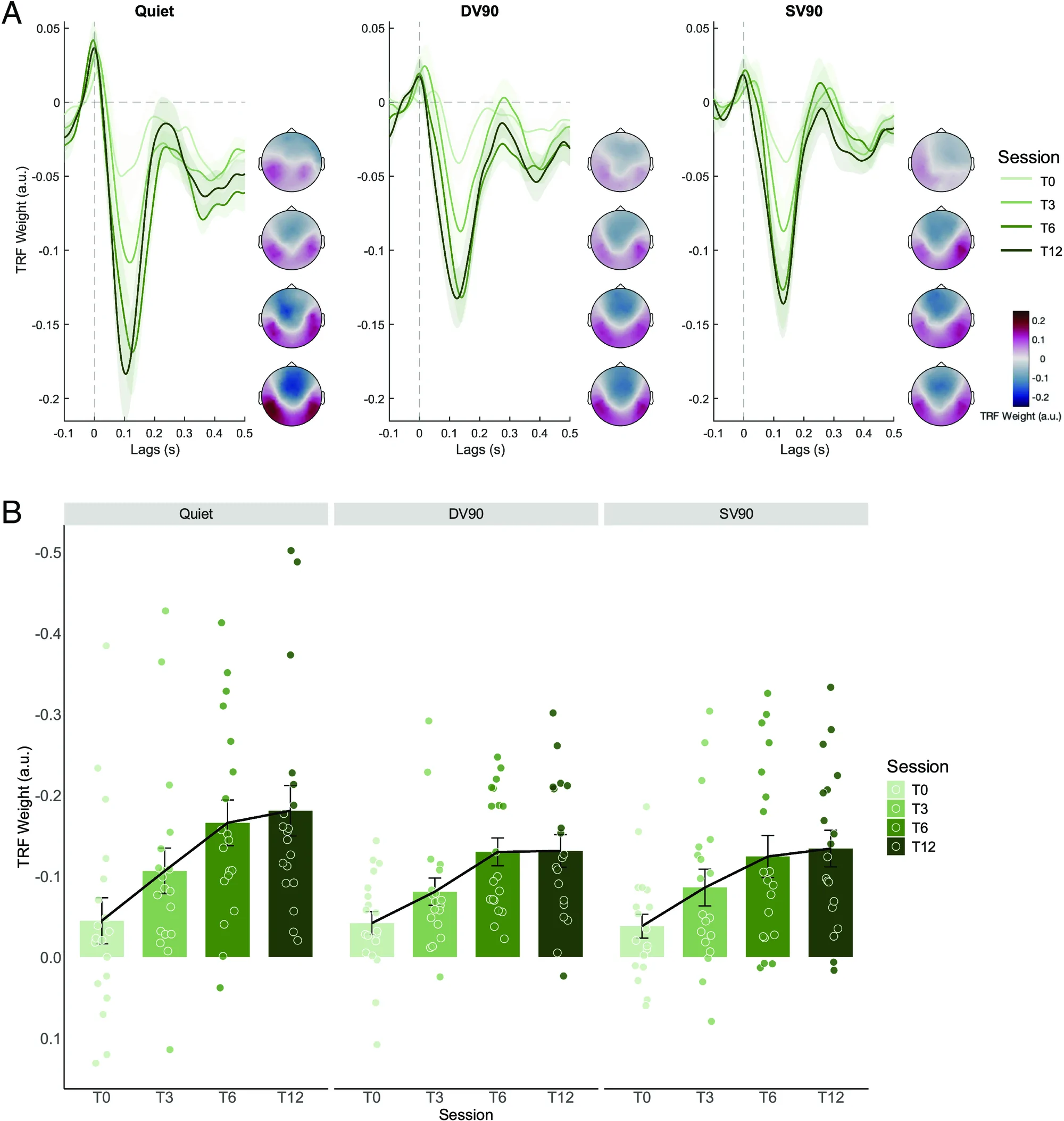
Longitudinal changes in target-related TRFs after CI activation. (*A*) Grand-average target-speech TRFs in the Quiet, DV90, and SV90 conditions at each session (T0, T3, T6, and T12), averaged across three fronto-central electrodes (*SI Appendix*, Fig. S2). Shaded bands indicate the SEM. Topographic maps show the scalp distribution of TRF activity at the negative peak for each session, ordered from top to bottom as T0, T3, T6, and T12. (*B*) Mean TRF weight extracted from a time window of ±8 ms around the grand-mean waveform peak latency, averaged across the same three fronto-central electrodes, for each listening condition and session. Each dot represents an individual participant. Bar plots show the group mean, and error bars indicate the SEM. Overlaid lines illustrate longitudinal changes in the group mean across sessions. More negative TRF weights indicate stronger target-speech tracking.

We first examined whether cortical tracking of the target speech stream changed during the first year after cochlear implantation across listening conditions. This analysis provided an index of how neural encoding of task-relevant speech evolved with CI experience. Target-speech TRF N1 mean amplitude changed significantly across sessions, *F*(3, 54) = 28.30, *P* < 0.001, $\eta_{G}^{2}=0.159$, but did not differ across listening conditions, *F*(2, 36) = 1.80, *P* = 0.18, $\eta_{G}^{2}=0.019$, and showed no condition × session interaction, *F*(6, 108) = 0.85, *P* = 0.53, $\eta_{G}^{2}=0.007$ (Fig. 3). Pairwise comparisons showed that N1 amplitude increased from T0 to T3 and continued to strengthen from T3 to T6, with no further significant change between T6 and T12 (*P* = 0.245). These findings indicate that cortical tracking of target speech strengthened during the first 6 mo of CI use and then plateaued, with a similar trajectory across listening conditions.

#### Target-speech TRF topographies changed across postimplant experience.

To determine whether longitudinal changes in target-speech TRFs involved changes in response topography in addition to response magnitude, we used topographic ANOVA (TANOVA). This analysis tests whether scalp maps differ in spatial configuration after normalization for overall amplitude and, therefore, can indicate changes in the relative contribution of neural generators over time. Topographic consistency tests (TCTs) confirmed the reliability of TRF topographies across participants for each condition and session, supporting the use of TANOVA (*SI Appendix*, Fig. S3).

**Fig. 4.**
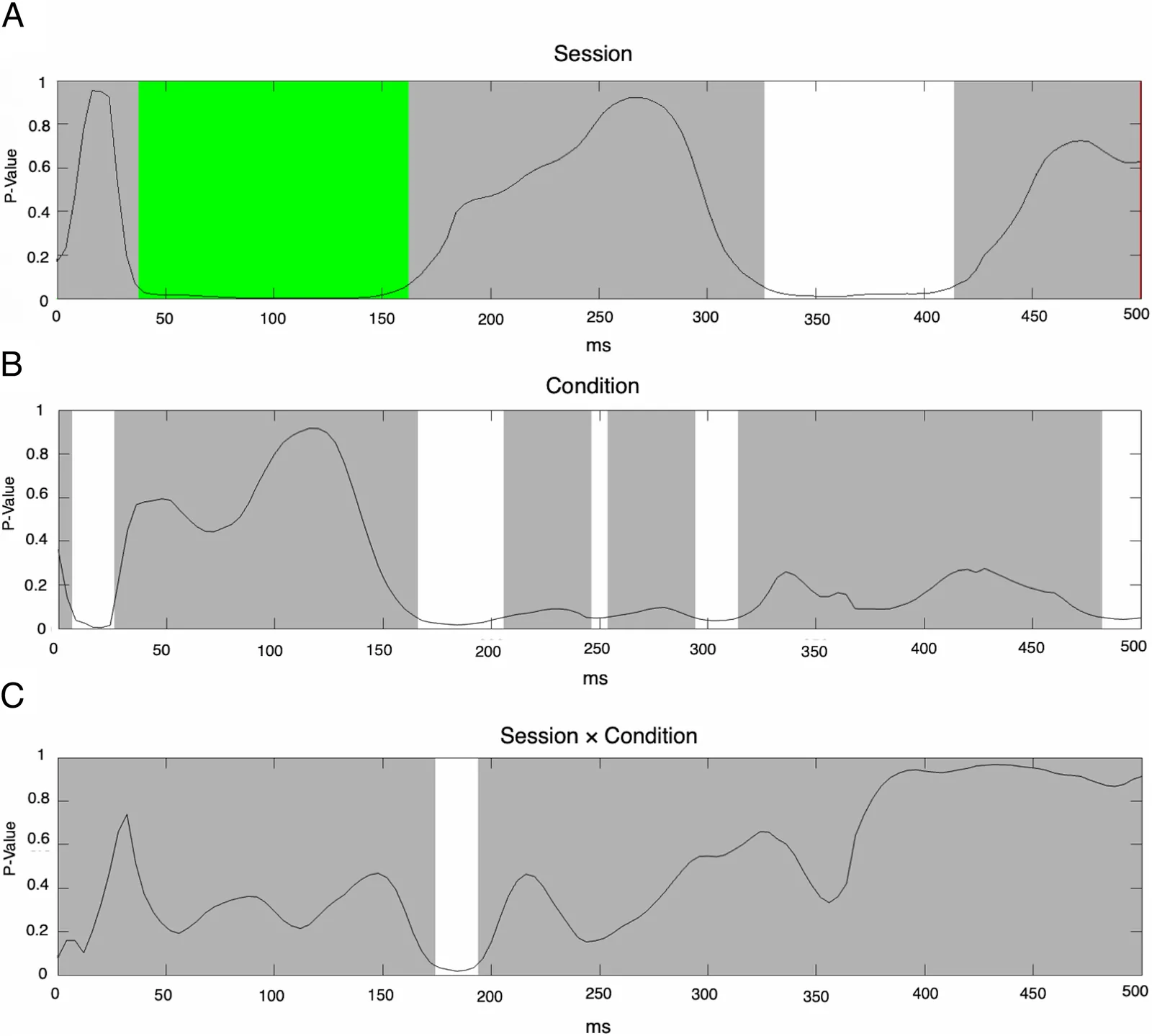
TANOVA *P*-value waveform across time. Gray shading indicates time points at which the point-wise TANOVA *P*-value was not significant. White intervals indicate latencies with point-wise TANOVA *P* < 0.05 before correction for multiple temporal comparisons. The green shaded interval marks the period that remained significant in the permutation-based global duration analysis and is therefore the interval interpreted in the main text. (*A*) TANOVA *P*-value waveform of the session effect. (*B*) TANOVA *P*-value waveform of the condition effect. (*C*) TANOVA *P*-value waveform of session × condition interaction.

TANOVA revealed a significant effect of session on target-speech TRF topography (global p-AUC = 0.031, global count *P* = 0.022), with significant topographic differences between T0 and later sessions in the early TRF time window (Fig. 4). Post hoc comparisons showed that T0 differed from T6, *P* = 0.048, and T12, *P* = 0.004, over approximately 40 to 160 ms lags, with differences most evident over fronto-central scalp regions. In contrast, there was no significant effect of listening condition and no condition × session interaction. These findings suggest that longitudinal changes in target-speech tracking were not limited to increased response magnitude but also involved changes in the spatial distribution of cortical responses with CI experience.

#### Selective tracking of target speech strengthened with CI experience.

**Fig. 5.**
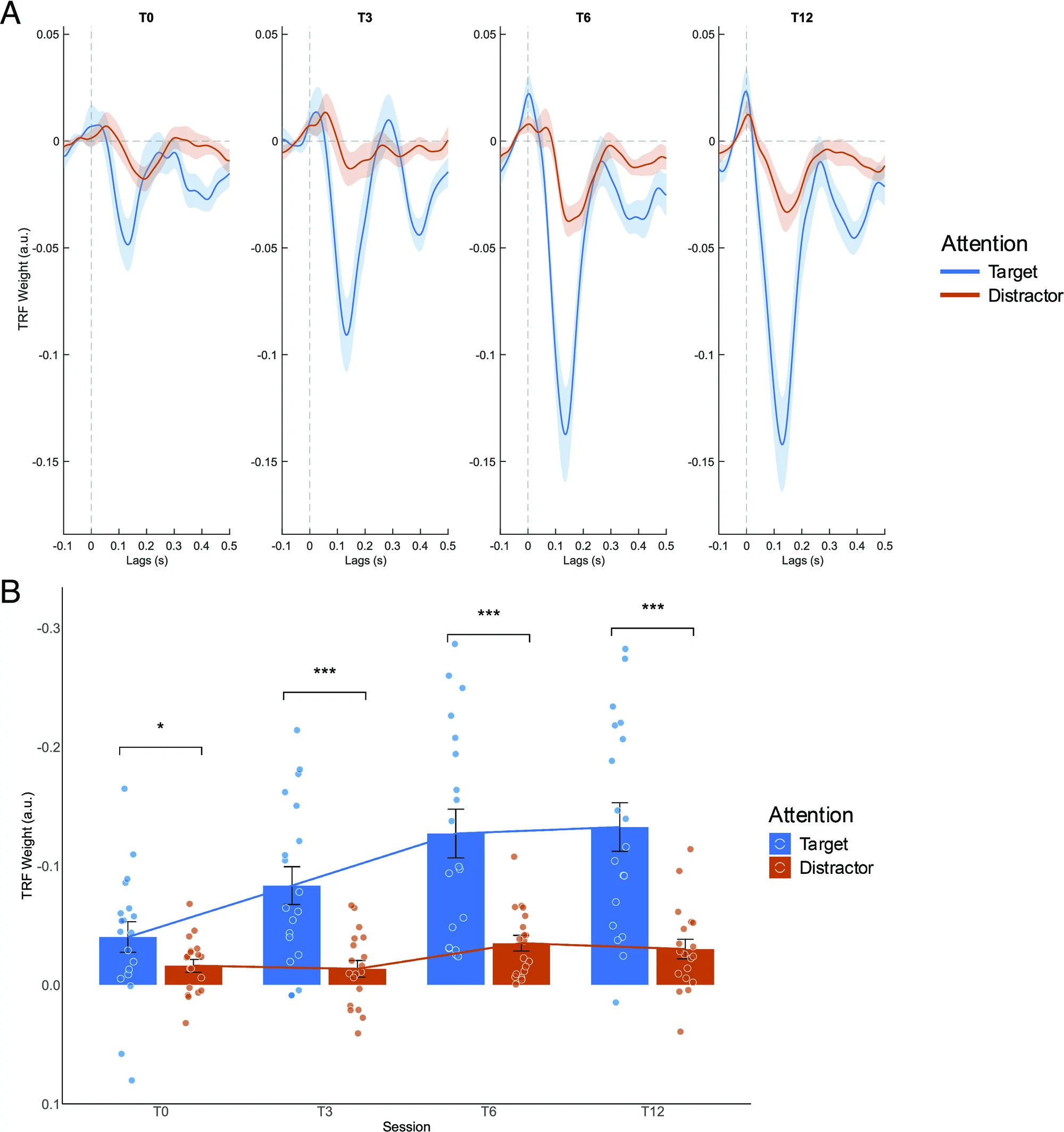
Longitudinal changes in target- and distractor-related TRFs. (*A*) Grand-average TRFs for the target and distractor speech streams at each session (T0, T3, T6, and T12), averaged across three fronto-central electrodes. Shaded bands indicate the SEM. (*B*) Mean TRF weights extracted from a ±8 ms window around the grand-mean waveform peak latency and averaged across the same three fronto-central electrodes, for the target and distractor streams at each session. Each dot represents an individual participant. Bar plots show the group mean, and error bars indicate the SEM. Overlaid lines illustrate longitudinal changes in the group mean across sessions. More negative TRF weights indicate stronger neural tracking. Horizontal brackets indicate within-session comparisons between target and distractor TRF weights; *P < 0.05, ***P < 0.001.

Having established that cortical tracking of the target stream strengthened over time, we next asked whether this longitudinal change reflected improved stream segregation of the task-relevant target from task-irrelevant distractors. We therefore compared mean TRF N1 amplitudes for target and distractor speech streams across sessions (Fig. 5). TRF responses were significantly stronger for target than for distractor speech, *F*(1, 18) = 33.96, *P* < 0.001, $\eta_{G}^{2}=0.286$, indicating preferential neural tracking of the target speech stream. TRF magnitude also changed significantly across sessions, *F*(3, 54) = 16.64, *P* < 0.001, $\eta_{G}^{2}=0.136$, increasing from early sessions and plateauing between T6 and T12.

Importantly, this longitudinal change differed between the target and distractor streams, as reflected by a significant attention × session interaction, *F*(3, 54) = 10.43, *P* < 0.001, $\eta_{G}^{2}=0.065$. Follow-up comparisons showed that target-speech tracking increased robustly from T0 to T3 and continued to strengthen through T6, with no further significant change between T6 and T12. In contrast, distractor-speech tracking showed a smaller increase over time, reaching significance only between T0 and T6 (*P* = 0.016) and between T3 and T6 (*P* = 0.003), with no significant differences for the remaining session pairs. Together, these findings indicate that the longitudinal increase in neural speech tracking was driven primarily by enhanced tracking of the target stream, consistent with improved selective encoding of task-relevant speech after cochlear implantation.

Exploratory analyses of TRF-N1 peak latency are reported in *SI Appendix*, Figs. S5 and S6. Briefly, target-related TRFs peaked earlier than distractor-related TRFs, and TRFs in the Quiet condition peaked earlier than those in competing-speech conditions; however, these latency effects did not change significantly across sessions.

#### Stronger target-speech tracking was associated with better behavioral outcomes.

**Fig. 6.**
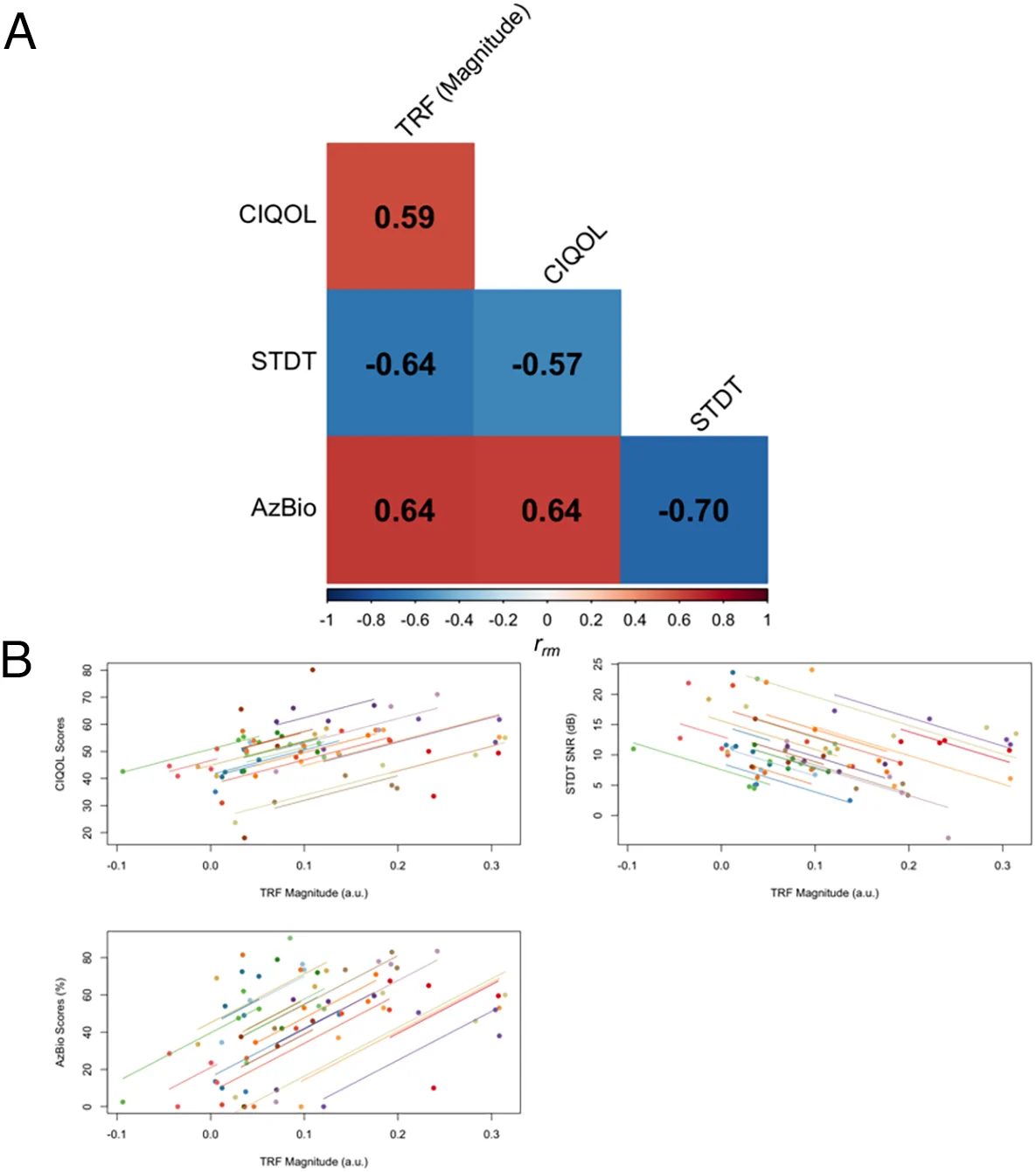
Repeated-measures correlation matrix among neural, behavioral, and self-reported outcomes. (*A*) Lower-triangular cells display repeated-measures correlation coefficients between target TRF magnitude (amplitude sign-flipped for visualization), CIQOL scores, STDT threshold SNR, and AzBio scores across longitudinal sessions. Values within each cell are the repeated-measures correlation coefficients, and all shown correlations were statistically significant (*P* < 0.05). Cell color indicates the direction and strength of the association, with blue representing negative correlations and red representing positive correlations; darker shading reflects stronger relationships. Higher TRF magnitude and lower STDT threshold SNR indicate better performance. (*B*) Repeated-measures correlation plots showing within-subject associations between TRF magnitude and behavioral outcomes across sessions. Each point represents one observation from a single participant at one session, and points/lines with the same color belong to the same participant. Solid lines indicate the repeated-measures correlation fit for each participant after accounting for between-subject variability. From *Left* to *Right* and *Top* to *Bottom*, panels show the associations between TRF magnitude and CIQOL scores, STDT threshold SNR, and AzBio percent correct. Higher TRF magnitude, AzBio scores, and CIQOL scores indicate better performance, whereas lower STDT threshold SNR indicate better speech-in-noisse performance.

Repeated-measures correlations were conducted to examine whether within-subject changes in neural speech tracking were related to changes in behavioral and functional outcomes across sessions (Fig. 6). Stronger TRF N1 responses were associated with better outcomes across measures: larger TRF magnitudes were related to higher CIQOL composite scores, better AzBio performance, and lower STDT thresholds (i.e., better spatial listening performance). These findings indicate that participants’ improvements in cortical tracking of attended speech were accompanied by gains in perceived quality of life, sentence recognition, and spatial speech-in-noise performance.

The behavioral measures were also strongly interrelated. Higher CIQOL scores were associated with better AzBio performance, and lower STDT thresholds, while better AzBio performance was associated with lower STDT thresholds. Together, these correlations suggest that neural, behavioral, and self-reported outcomes changed in a coordinated manner over the first year of CI use.

#### Supplementary model comparison of neural and behavioral predictors.

To test whether neural speech tracking explained variance beyond behavioral measures, we conducted a supplementary incremental model comparison of linear mixed-effects models predicting CIQOL composite scores. The baseline model included session, AzBio sentence recognition, and STDT threshold as fixed effects, with participant as a random intercept. The full model additionally included TRF amplitude. Adding TRF significantly improved model fit, χ²(1) = 7.80, *P* = 0.005, reduced both the Akaike Information Criterion (AIC; lower values indicate better fit after penalizing model complexity, ΔAIC = −5.80), and the Bayesian Information Criterion (BIC; a more conservative measure of model fit that penalizes model complexity, with lower values indicating better fit, ΔBIC = −3.65), and increased marginal R² from 0.440 to 0.514. These results suggest that neural tracking of continuous speech captured outcome-relevant variance that was not fully explained by behavioral measures alone.

## Discussion

### Time Course of Postimplant Adaptation.

The current study investigated the behavioral and neural trajectories of CI adaptation. Across measures, changes were most pronounced in the early postactivation period and stabilized by approximately 6 mo. This pattern suggests a rapid–then–slower trajectory rather than linear improvement over the first year. Compared with the previous literature reporting a common 3-mo plateau (4, 21, 29, 32), our findings suggest that the apparent timing of stabilization depends on the processing demands of the outcome measure. Mechanistically, this trajectory suggests that postimplant adaptation involves partly separable processes: early access to the temporal structure of the CI signal, followed by slower recalibration of auditory, attentional, and linguistic systems that support speech understanding in complex environments. At the neuronal population level, larger TRF weights likely reflect stronger or more temporally aligned cortical responses to speech-envelope fluctuations (36, 37). Because cortical speech-envelope representations can be amplified and precisely phase-locked (38), the observed longitudinal increase is consistent with experience-dependent strengthening of cortical speech tracking after implantation.

The relatively prolonged trajectory observed for speech TRFs may be partly explained by the naturalistic listening demands of the present paradigm. Earlier neurophysiological studies have largely relied on simple tonal or highly controlled auditory stimuli (21, 30–32, 39), which are useful for isolating basic auditory processes but do not fully engage real-world listening processes (40, 41). In contrast, our continuous speech selective-attention paradigm required listeners to encode the target speaker while segregating competing auditory streams, sustaining attention, and suppressing irrelevant speech (42–46). Thus, continued changes in speech TRFs may reflect plasticity in distributed auditory–attention networks, rather than recovery of early sensory responsiveness alone (47, 48).

### Strengthening of Selective Attention to Speech.

A central finding of the present study was that target and distractor speech tracking followed distinct longitudinal trajectories after CI activation. Target-speech tracking showed earlier and larger increases than distractor speech tracking, and the target–distractor difference was smaller at T0 and T3 than at T6 and T12. Because the increase was larger for target than for distractor speech, the effect is unlikely to reflect a nonspecific increase in auditory responsiveness. Instead, it suggests progressively stronger attention-dependent modulation of cortical gain, whereby neuronal populations aligned with the target speaker are preferentially enhanced relative to those aligned with task-irrelevant distractor speech (38, 42, 49–52). At the neuronal population level, this increasing separation may reflect sharper response selectivity: cortical ensembles aligned with the target stream become more strongly phase-locked to target-envelope fluctuations, whereas competing streams are represented at lower gain or more selectively gated.

One possible account of this emerging target–distractor separation is that CI experience changes how listeners allocate processing resources over time. Because CIs provide spectrally reduced and frequency-shifted input, early postactivation speech signals may be poorly matched to central speech representations established through prior acoustic hearing (53–57). From an Ease of Language Understanding (ELU) perspective (58–60), this mismatch may increase reliance on effortful processing, with cognitive resources initially directed toward basic signal interpretation and lexical access rather than efficient segregation of target and competing speech. The smaller early target–distractor difference may therefore reflect less selective cortical gain across competing streams while the system is still learning which cues are informative for speech recognition through the implant. With increasing CI experience, listeners may develop a more efficient mapping between electric input and higher-order speech representations, allowing processing to shift toward more automatic target selection and distractor suppression.

The behavioral findings show a broadly consistent but not identical time course. STDT performance suggested that the behavioral benefit of spatial separation became evident by approximately 6 mo postactivation. This does not imply that spatial cues were unavailable earlier; rather, additional CI experience may be needed before spatial information is integrated effectively enough to support measurable improvements in competing-speech performance. Neural target–distractor differentiation may therefore provide a more sensitive index of emerging selective cortical processing, whereas behavioral spatial-separation benefits may appear only once these neural changes become sufficiently robust to affect task performance. This interpretation is consistent with frameworks of auditory scene analysis and object-based attention, which emphasize that complex listening depends not only on sound encoding but also on auditory object formation, target selection, and distractor suppression (33–35, 43, 61).

The delayed increase in distractor tracking should not be interpreted simply as poorer suppression of irrelevant speech. In everyday listening, task-irrelevant sounds can quickly become relevant, requiring listeners to monitor background streams and reorient attention when needed. The later increase in distractor tracking may therefore reflect the emergence of more stable cortical representations of background speech objects, supporting flexible attention allocation after implantation. Monitoring background speech may be an important but underexamined component of listening after cochlear implantation. Because ignored sounds can become relevant in everyday environments, rehabilitation may depend not only on enhanced target tracking but also on maintaining background representations that support flexible attention switching. Future studies should directly examine how background monitoring and attention reorientation develop after implantation.

### Functional Relevance of Neural Speech Tracking.

Although behavioral and neural measures did not follow identical trajectories, repeated-measures correlations indicated that target-speech tracking was associated with functional performance within individual CI users. Specifically, sessions with stronger target-speech tracking were associated with better speech recognition, better spatial speech-in-noise performance, and higher hearing-related quality of life. This suggests that longitudinal increases in target-speech tracking may index a functionally relevant adaptive process that covaries with improvements in speech understanding and self-reported listening outcomes. Importantly, because these associations were observed across measures involving different speakers and listening contexts, they suggest that TRF changes were not merely task-specific fluctuations in the EEG paradigm but may reflect broader changes in how speech is neurally encoded during postimplant adaptation. This interpretation is consistent with prior work showing that cortical speech tracking is modulated by selective attention and reflects the neural representation of attended speech (38, 49, 62). Supplementary model comparisons further suggested that TRF amplitude captured outcome-relevant variance beyond conventional behavioral measures, although this should be interpreted as incremental explanatory value rather than established clinical prediction or mechanistic specificity. Consistent with this interpretation, supplementary analyses of listening effort showed that perceived effort varied by condition and was associated with TRF-N1 amplitude, suggesting that neural speech tracking may also relate to perceived listening demands during naturalistic speech listening (*SI Appendix*, Fig. S4 and Table S2).

### Implications.

The present findings have implications beyond characterizing CI outcomes alone. Clinically, characterizing adaptation in a naturalistic listening context may help provide patients and families with a more realistic expectation of the pace of postimplant change, which CI candidates identify as an important consideration in decision-making (3, 63). The observed trajectory should not be interpreted as a validated prognostic tool, but it may provide preliminary context for understanding recovery over time.

Mechanistically, the findings suggest that successful CI adaptation depends not only on peripheral access to sound but also on plasticity in cortical speech encoding, selective attention, and auditory scene analysis. By measuring behavioral, patient-reported, and neurophysiological outcomes in the same individuals at matched time points, the present study provides an integrated account of adaptation across levels of analysis. Behavioral improvement alone does not specify underlying neural mechanisms, whereas neural change alone does not establish functional relevance. The convergence between stronger target-speech tracking, improved behavioral performance, and better quality of life suggests that cortical speech tracking captures a functionally meaningful component of postimplant adaptation.

Future work should test whether early neural, behavioral, or patient-reported measures can predict individual CI outcomes and identify patients who may benefit from more individualized rehabilitation. Such studies will require larger, multicenter longitudinal cohorts with formal predictive modeling, and should consider individual differences that shape recovery, including duration of deafness, residual hearing, and prior auditory experience (22, 64). Future studies may also extend the TRF framework to audiovisual paradigms, given the importance of visual speech cues for many individuals with hearing loss (65, 66), and to reduced-electrode systems such as cEEGrid or in-ear EEG to support repeated assessment in clinical or ambulatory settings (67–69).

### Limitations.

The absence of a longitudinal control group is a notable limitation of the study. Although different audiobook segments were used across sessions to reduce repeated-stimulus effects, the design cannot fully separate implant-related adaptation from nonspecific longitudinal factors such as task familiarity, repeated testing, or measurement stability. Thus, the within-participant design characterizes postimplant trajectories but does not establish that the observed changes were specific to CI experience alone.

Second, the discrete sampling schedule limits conclusions about the precise timing of stabilization. Although several behavioral and neural measures appeared to plateau by approximately 6 mo, this should not be interpreted as evidence that adaptation stabilizes exactly at that time. More frequent follow-up assessments, particularly during the first year after activation, would better clarify the temporal dynamics of postimplant adaptation.

Finally, TRF measures likely reflect a combination of sensory encoding, attention, and higher-order linguistic processing, limiting attribution to a single mechanism. The neuronal-level interpretations proposed above should therefore be understood as population-level inferences from scalp EEG, not as direct evidence of single-neuron activity or specific synaptic mechanisms. Larger TRF weights may reflect stronger cortical gain, improved temporal synchrony, reduced response variability, changes in neural generator configuration, or a combination of these processes (36–38). Because responses to simple transient sounds were not recorded, we also cannot directly compare the adaptation of early auditory responses with continuous speech tracking. Future studies that combine simple and naturalistic speech measures in larger samples would help clarify distinct stages of cortical adaptation and improve generalizability.

## Conclusion

In conclusion, the present study shows that adaptation to cochlear implantation follows a broadly consistent trajectory across behavioral, patient-reported, and neural measures. By combining longitudinal assessment with an ecologically valid continuous-speech selective-attention paradigm, we provide a more functionally relevant account of postimplant recovery. Across levels of analysis, the greatest changes were observed during the early months after activation, followed by relative stabilization by approximately 6 mo postactivation. The increasing neural differentiation between target and distractor speech further suggests progressive refinement of speech processing under complex listening conditions. Together, these findings highlight the importance of studying adaptation under realistic listening demands and establish an integrative framework for future longitudinal research on sensory rehabilitation.

## Materials and Methods

### Participants.

Participants were adult CI users who were native English speakers. All participants were unilateral CI users with no history of neurological disorders or major psychiatric conditions. Individuals with single-sided deafness or electric–acoustic stimulation were excluded to minimize potential confounding effects. Eligible participants were contacted and provided written informed consent to participate in the study. This study was approved by the Research Ethics Board at Sunnybrook Health Sciences Centre (REB #474-2016) and conducted in accordance with the Declaration of Helsinki.

Twenty-one adult CI users were recruited from the Department of Otolaryngology at Sunnybrook Health Sciences Centre. Two participants withdrew from the study, resulting in a final sample of 19 participants (9 males, 10 females). Participants ranged in age from 29 to 79 y (M = 64.6, SD = 12.7) and had an average of 26.3 y of hearing loss (SD = 13.6). Residual hearing calculated as the average pure tone audiometry between 0.5 kHz and 4 kHz was 85.4 dB (SD = 15.7). Ten participants received their CI in the left ear. Additional demographic and clinical characteristics are summarized in Table 1.

**Table 1.** Participant demographics

ID	Age (years)	Sex	Duration of hearing loss (years)	Residual hearing PTA (dB HL)^*^	Implant side	Etiology	CI brand
CI01	62	F	6	63.75	Left	Progressive SNHL—Idiopathic	MED-EL
CI02	65	F	40	58.75	Left	Progressive SNHL—Idiopathic	AB
CI03	64	M	43	102.5	Left	Otosclerosis	MED-EL
CI04	44	M	17	95	Right	Noise exposure	AB
CI05	55	M	16	91.25	Left	Noise exposure	AB
CI06	71	M	24	85	Left	Progressive SNHL— Idiopathic	MED-EL
CI07	29	F	14	106.25	Right	Progressive SNHL—Idiopathic	MED-EL
CI08	78	M	39	76.25	Left	Progressive SNHL—Idiopathic	MED-EL
CI09	68	M	18	75	Left	Progressive SNHL—Idiopathic	AB
CI10	79	F	30	87.5	Right	Progressive SNHL—Idiopathic	MED-EL
CI11	76	M	8	76.25	Right	Progressive SNHL—Idiopathic	AB
CI12	60	F	54	95	Right	Progressive SNHL—Idiopathic	MED-EL
CI13	50	F	33	72.5	Left	Progressive SNHL—Idiopathic	AB
CI14	65	F	15	71.25	Left	Progressive SNHL— Idiopathic	Cochlear
CI15	70	F	42	87.5	Right	Progressive SNHL—Idiopathic	AB
CI16	76	F	40	102.5	Right	Progressive SNHL—Idiopathic	MED-EL
CI17	68	F	19	63.75	Right	Progressive SNHL—Idiopathic	AB
CI18	73	M	18	105	Right	Progressive SNHL—Idiopathic	MED-EL
CI19	74	M	24	107.5	Left	Progressive SNHL—Idiopathic	MED-EL

^*^Pure-tone average (dB HL).

### Procedure.

Participants were tested at CI activation date (T0), 3 mo after CI activation (T3), 6 mo after activation (T6), and 12 mo after activation (T12). These time points were chosen to align with commonly used follow-up intervals in standard practice, allowing more direct comparison of the present findings with clinical findings and previous studies. A preimplantation recording was not obtained because the study was designed to examine adaptation after the onset of CI use. Given the difference between preimplant acoustic hearing and postimplant electrically mediated hearing, preimplant measures would not provide a directly comparable baseline for longitudinal assessment of CI adaptation. Therefore, the activation session (T0) served as the baseline time point.

Participants completed four tasks in each session: CIQOL, AzBio, STDT, and audiobook listening. All tasks were performed in a sound-attenuated and electrically shielded booth with minimal reverberation.

### CIQOL Survey.

The CIQOL Profile is a 35-item questionnaire developed specifically for CI users (70). It assesses the impact of hearing across six domains: communication (receptive and expressive abilities in various situations), emotion (the influence of hearing on emotional well-being), entertainment (clarity and enjoyment of media such as TV, radio, and music), environmental (recognition and localization of environmental sounds), social (participation in group interactions and social activities), and listening effort (the effort and fatigue associated with listening). Responses are recorded on a five-point scale (never, rarely, sometimes, often, always), with each option assigned a value from one to five, though some items are reverse-scored. After adjusting for reverse coding, scores within each domain are summed and then converted to an interval-scale score based on item response theory (70). Final scores range from 0 to 100, with higher values reflecting better self-reported functioning. A composite score was computed for each participant by averaging the scores of all domains.

### AzBio Sentence Test.

The AzBio sentence test (71) served as the clinical speech perception measure. Participants were positioned approximately 1 m from a loudspeaker located directly in front of them at ear level. They were asked to repeat 20 sentences in quiet (60 dB SPL) and 20 sentences in 10-talker babble noise (at +5 dB SNR). During each trial, participants repeated the sentence they heard, and responses were scored by counting the number of words correctly reported. Percent-correct scores for each sentence were calculated by dividing the number of correctly repeated words by the total number of words presented. Sentence scores were then averaged to provide an overall estimate of speech perception performance for the given listening condition.

### STDT.

The STDT consisted of a series of three spoken digits produced by a female speaker of standard American English (40, 72–75). A scale factor was applied to equalize the root mean squared amplitude of all digits.

During stimulus presentation, digits were played with an onset-to-onset interval of 1,195 ms. The digits in each trial were selected pseudorandomly, ensuring that no digit was repeated and that no ascending or descending numerical sequences occurred. Digits were presented in the same order as they were recorded within each triplet.

All stimuli were delivered through an array of five loudspeakers, with participants seated at the center. Each loudspeaker was positioned 1 m from the center, at azimuths of 0°, ±45°, and ±90°. The center of each speaker cone was 1 m above the floor. Target digits were always played from the front (0°) speaker at 65 dB SPL, measured at the center of the array at ear level. Distractor digits were played from either the ±45° or ±90° speakers. SNR decreased by 2 dB if a participant correctly reported the three target digits; otherwise, the SNR increased by 2 dB. At the end of each block, we calculated the STDT threshold (mean of the last 20 SNR presentations), which indicated the SNR required for the participant to identify the target digits with 50% accuracy. Participants completed this task under four conditions: same-voice distractors at ±45° (SV45), same-voice distractors at ±90° (SV90), different-voice distractors at ±45° (DV45), and different-voice distractors at ±90° (DV90).

Same-voice distractors were created so that the target and distractor streams shared the same talker identity. Different-voice distractors were created by shifting the target talker’s pitch by three semitones using STRAIGHT software (76). This manipulation ensured that the different-talker voice was distinguishable from the target voice (77, 78). All digits were processed in MATLAB R2009b (The MathWorks, Natick, MA), and stimulus delivery was controlled using a Tucker-Davis Technologies (TDT) RX8 Processor. Non-CI ears were occluded during listening.

### Naturalistic Listening Paradigm.

Participants listened to excerpts from the audiobook Diary of a Wimpy Kid under three listening conditions: Quiet, DV90, and SV90. Each condition lasted approximately 15 min. In the Quiet condition, the target story was presented alone. In the DV90 and SV90 conditions, two background distractor streams, each comprising story segments from a different series, were presented from speakers at ±90° azimuth, while the target stream was always delivered from the front (0° azimuth) speaker. The SV90 condition used distractors spoken by the same talker as the target, whereas the DV90 condition used the same different-voice manipulation described for the STDT. The target story was presented at 65 dB SPL, and each task-irrelevant story stream was presented at 60 dB SPL. Task-irrelevant stories were never used as target materials.

Before listening to the story, participants were instructed to pay close attention to the target story, as they would be tested with five comprehension questions at the end of each condition. Non-CI ears were occluded during listening.

### Recording and Preprocessing.

EEG data were recorded using a 64-channel actiCHamp system (Brain Products GmbH, Munich, Germany) at a sampling rate of 2,000 Hz. Participants wore EEG caps with electrodes positioned according to the international 10 to 20 system, with the Cz electrode placed at the vertex, defined as the intersection of the nasion-to-inion and tragus-to-tragus midpoints (18, 79, 80). A reference electrode located slightly anterior to the vertex on the midline served as the online reference, and the ground electrode was placed midway between the nasion and the vertex. Electrodes located near or over the CI magnet and coil on the implanted side were excluded from recording, typically resulting in the removal of 2 to 4 electrodes per participant.

Raw EEG data were processed in Brain Vision Analyzer (Brain Products, Gilching, Germany), band-pass filtered between 0.1 Hz and 40 Hz with a second-order Butterworth filter and downsampled to 250 Hz. Noisy channels and segments containing excessive artifacts were excluded from the data. Independent Component Analysis was then applied to the EEG to identify stereotypical physiological artifacts (e.g., eye blinks, eye movements, and cardiac activity) and technical artifacts (e.g., electrode pops and line noise) based on component topographies, time courses, and spectral properties. Artifact-related components were manually removed after visual inspection of their topographies. Subsequently, excluded channels were interpolated using spherical spline interpolation from neighboring electrodes (81).

The target audio stream was recorded concurrently with the EEG via an auxiliary channel to ensure precise temporal alignment between the auditory stimulus and EEG recordings. Offline, the recorded signal was aligned to the original soundtrack by computing the cross-covariance, enabling identification of the exact stimulus onset using custom MATLAB scripts. In the multitalker conditions, two distractor streams were presented concurrently from the lateral loudspeakers. Due to limited auxiliary channel availability during EEG acquisition, these distractor streams were not recorded online via auxiliary channels. Instead, the original distractor audio files were aligned offline using the stimulus onset identified from the target auxiliary channel and appended to the EEG dataset as two additional time-aligned stimulus channels. For the TRF analyses, the target stream and the two distractor streams were modeled as separate stimulus regressors, allowing target- and distractor-related TRFs to be estimated separately.

Broadband speech envelopes were extracted by taking the absolute value of the Hilbert transform of the aligned audio signal. The resulting envelopes were downsampled to match the EEG sampling rate and appended to the EEG data for subsequent analyses.

### CI Artifact Suppression.

We employed second-order blind identification (SOBI) to minimize CI-related contamination in EEG data from CI participants (18, 42, 82, 83). This blind source separation method uses second-order statistics to separate temporally correlated sources (82). SOBI was applied to each participant’s continuous EEG data, and artifact-related components were visually identified based on their topographical centroids and the implantation side during the listening task. A total of 0 to 2 components were removed per participant. The cleaned EEG data and aligned audio streams were then imported into FieldTrip (84) and rereferenced to the average reference before further processing for TRF analysis.

### Speech Neural Tracking: TRF.

We used the mTRF toolbox 2.0 (36) in MATLAB to generate TRF models. Both speech-envelope predictors and EEG signals were filtered using a 1 to 20 Hz, second-order, zero-phase Butterworth bandpass filter. Following filtering, audio signals and EEG data were z-scored for each participant prior to TRF modeling. Time lags ranging from −100 to 500 ms were selected for analysis.

To reduce overfitting, the regularization parameter λ was optimized via cross-validation. Continuous EEG data were segmented into 60-s epochs, and the mTRFcrossval function was used to evaluate prediction accuracy across a logarithmic range of λ values from 2^−5^ to 2^15^. This function implements leave-one-out N-fold cross-validation, in which TRFs are estimated from all epochs except one and the resulting encoding model is used to predict the EEG response for the held-out epoch. Prediction accuracy was quantified using Pearson’s correlation coefficient between the predicted and recorded EEG signals for each channel. Correlation coefficients were averaged across participants, channels, and conditions for each λ value, and the λ yielding the highest average correlation was selected as the group-level regularization parameter. Prediction accuracies for each condition and attention streams were reported in *SI Appendix*, Fig. S1 and Table S1.

Using the selected group-level λ, final condition-specific TRF models were then trained for each participant and session using all available data from that condition. For each channel, TRF weights were baseline-corrected by subtracting, at all time points, the mean amplitude during the −100 to −4 ms prestimulus baseline. The final baseline-corrected TRF models were exported in BESA avr format for subsequent statistical analysis.

### Statistical Analysis.

An a priori power analysis indicated that eight participants would be required to detect a within-subject main effect of session across four time points at α = 0.05 with 80% power, assuming a conservative nonsphericity correction (ε = 0.60). The analysis was based on an effect size derived from a longitudinal study (32) examining N1 amplitude changes across session ($\eta_{p}^{2}=0.25$; *f* = 0.58).

For behavioral measures (AzBio speech recognition scores, STDT thresholds, and CIQOL composite scores), repeated-measures ANOVAs were conducted with session as a within-subject factor (four levels: T0, T3, T6, T12). For the STDT, distractor location (45° vs. 90° spatial separation) and voice identity (DV, SV) were included as additional within-subject factors. For neural measures, TRF N1 mean amplitude was analyzed using repeated-measures ANOVAs with condition (DV90, SV90, Quiet), or attention (target stream vs. distractor streams) and session as within-subject factors. Mean TRF amplitude was extracted from a time window of ±8 ms around the grand-mean waveform peak latency, averaged across three fronto-central electrodes (*SI Appendix*, Fig. S2).

When the assumption of sphericity was violated, as assessed by Mauchly’s test, Greenhouse–Geisser corrections were applied to the degrees of freedom. Effect sizes are reported as generalized eta squared ($\eta_{G}^{2}$). Significant main effects were followed by pairwise comparisons, with FDR correction applied to control for multiple comparisons.

To examine relationships between neural and behavioral outcomes across sessions, repeated-measures correlations were performed using the rmcorr approach (85) in R (86), which accounts for the nonindependence of observations within participants. Correlations were computed between TRF N1 amplitude and AzBio scores, STDT thresholds, and CIQOL composite scores.

Scalp-level TRF topographies were analyzed in RAGU (Randomization Graphical User Interface) (87), a standalone MATLAB-based graphical user interface. We used a permutation-based approach to test whether sessions and conditions differed in the spatial distribution of scalp activity independent of overall response strength. TCTs were first used to identify time periods with consistent scalp configurations across participants. At each time point, the global field power of the group-average map was compared with a null distribution generated by repeatedly permuting electrode values across channels separately within each participant and recalculating the group-average map.

TANOVA was then used to assess differences in scalp distributions across conditions and sessions. Because different scalp topographies may reflect differences in underlying neural generators, this analysis assessed whether the spatial configuration of TRF responses changed over time in newly implanted CI users. Before analysis, scalp maps were L2-normalized to isolate spatial differences independent of amplitude. TANOVA quantified global dissimilarity between condition-specific maps, and significance was evaluated using 5,000 random permutations of condition labels across participants. To control for multiple comparisons across time, overall significance was assessed using three global permutation-based metrics: Global Count Statistics, Global p-AUC Statistics, and Global Duration Statistics. These indices assess whether the number, overall strength, and temporal extent of significant effects exceed chance levels, providing complementary evidence of topographic differences while controlling the overall false-positive rate.

Statistical significance was set at α = 0.05 for all analyses using two-tailed tests. All statistical analyses were conducted in R (version 4.5.1) and MATLAB (2023b). Scalp topographic analyses were performed using RAGU (87).

## Supplementary Material

Appendix 01 (PDF)

## Data Availability

Custom MATLAB scripts for audio alignment, SOBI-based CI artifact suppression, and TRF modeling are available at https://github.com/shiminmo/CIL-longitudinal-TRF-code.git (88). Preprocessing steps performed using commercial or third-party software are described in Materials and Methods section to support reproducibility. The full raw dataset (including participant-level EEG data) is not publicly shared due to confidentiality considerations. De-identified analysis-level participant data underlying the statistical results, including the behavioral data and the measures extracted from the neural recordings have been deposited in https://osf.io/j3ea4 (89).
