# Supplementary material for: Longitudinal adaptations in neural and behavioral systems following hearing restoration using cochlear implants
Source: Proc Natl Acad Sci U S A. 2026 Sep 28;123(40):e2613077123. doi: 10.1073/pnas.2613077123 (PMC13643281; doi:10.1073/pnas.2613077123)
Supplement: Supplementary file 1 [file pnas.2613077123.sapp.pdf]

PNAS

Supporting Information for

Longitudinal Adaptations in Neural and Behavioural Systems Following Hearing Restoration Using Cochlear Implants

Shimin Mo, Claude Alain, Andrew Dimitrijevic

*Corresponding author: Andrew Dimitrijevic, andrew.dimitrijevic@utoronto.ca

This PDF file includes:

Supporting text
Figures S1 to S6
Tables S1 to S2

Fig. S1. TRF prediction accuracy across sessions. Asterisks indicate significantly greater prediction accuracy for target than for distractor speech within each session, all p < .001. Error bars indicate the standard error of the mean.

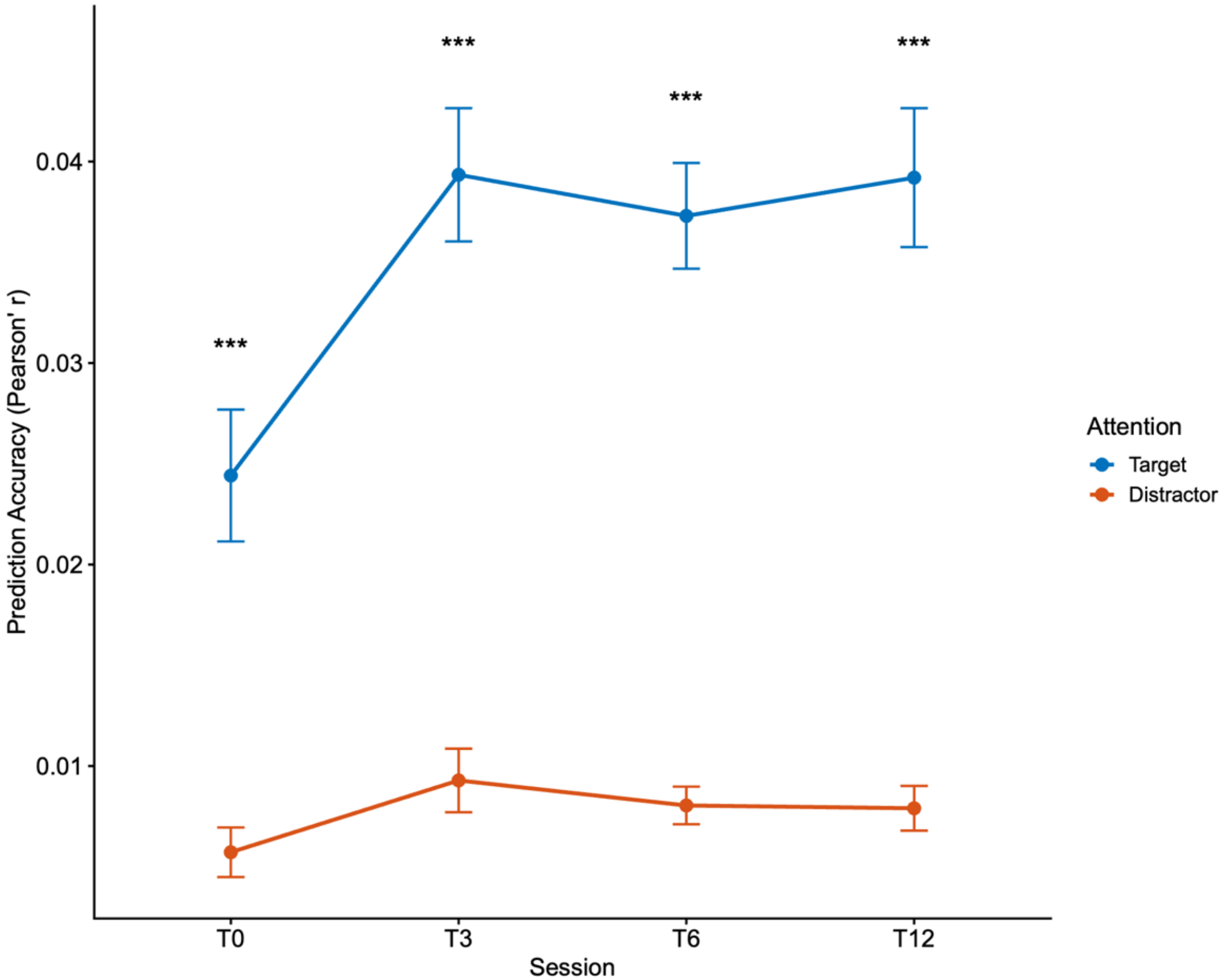

Fig. S2. Frontal-central channels used in the analysis. The three frontocentral EEG channels selected for TRF analysis are highlighted.

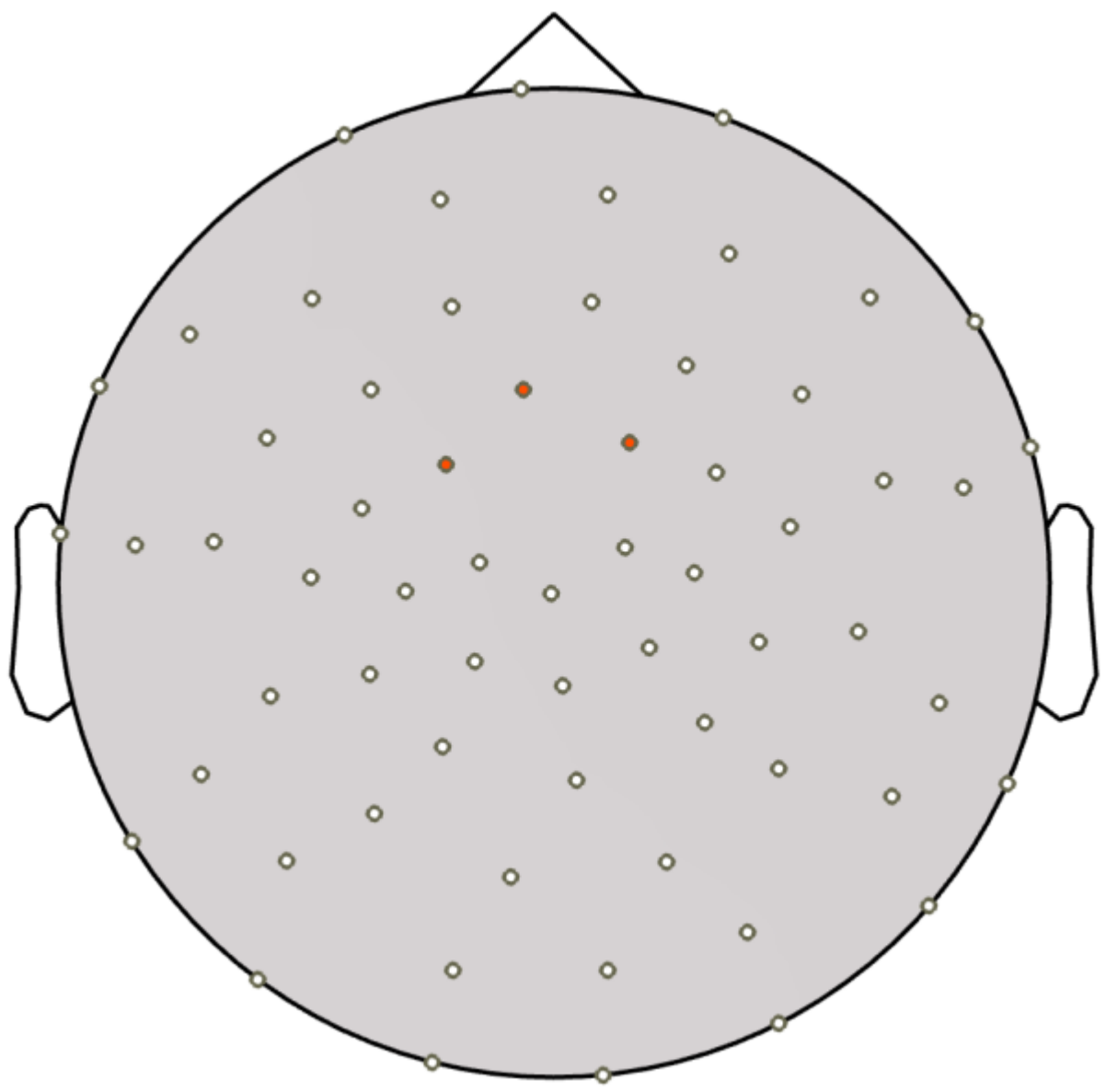

Fig. S3. Topographic Consistency Test (TCT) Results. TCT showed consistent neural activity across all conditions and sessions. The red line marks the significance threshold at $p = 0.05$. Gray shading denotes time points that did not reach significance, with the height of the shading reflecting the TCT p-value; unshaded (white) regions indicate significant time points ($p < 0.05$).

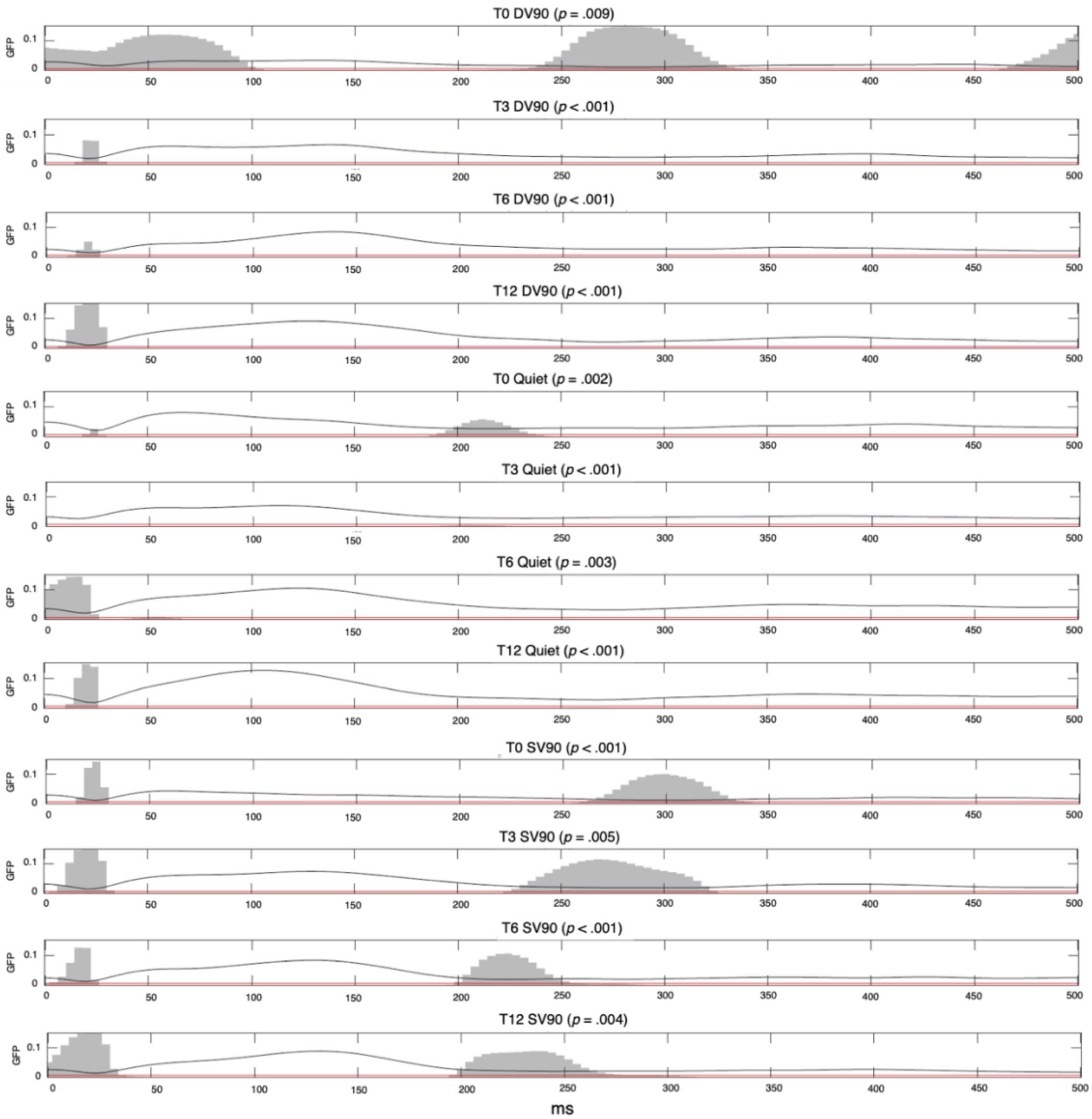

Fig. S4. Subjective Listening Effort Ratings

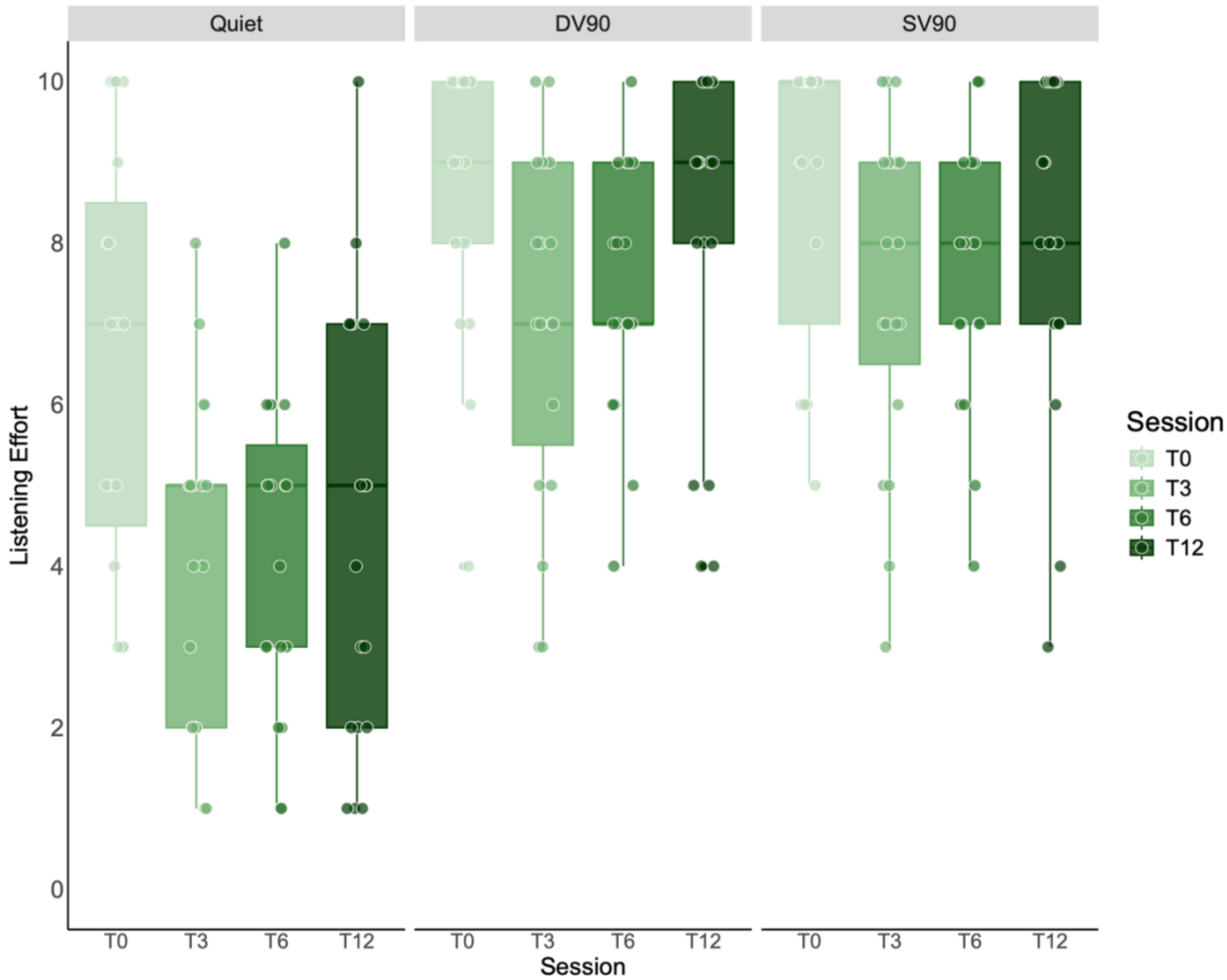

After each audiobook listening condition, participants verbally rated their listening effort on a 1–10 scale. Listening effort was defined verbally by the experimenter and displayed on a printed sheet as "the mental demand required to understand the spoken numbers," with "1" representing the least possible effort and "10" the greatest. Participants were instructed to respond "not applicable" if they felt they had not exerted effort due to lapses in attention. During task instruction, experimenters carefully distinguished listening effort from perceived difficulty. Specifically, participants were told not to rate how "hard" the condition seemed, but rather to report the effort they felt they were applying while listening to the audiobook.

A linear mixed-effects model was used to examine whether TRF-N1 amplitude was associated with listening effort, while accounting for session and listening condition, with participant included as a random intercept. Model output is shown in Table S2.

Listening effort was significantly associated with TRF-N1 amplitude after accounting for session and listening condition. Specifically, less negative TRF-N1 amplitudes were associated with higher listening effort ratings, suggesting that weaker neural tracking responses were linked to greater perceived effort during audiobook listening. Listening effort also varied across sessions, with significantly lower effort reported at T3 and T6 relative to T0, although the difference between

T12 and T0 was not significant. In addition, both spatial speech conditions were associated with substantially higher listening effort than Quiet, consistent with the increased perceptual demands imposed by competing speech. Together, these findings suggest that listening effort was sensitive to both the external listening context and related to neurophysiological response strength, with greater effort reported in more challenging conditions and when TRF-N1 responses were reduced.

Fig. S5. TRF-N1 Latency by Conditions. Error bars indicate the standard error of the mean.

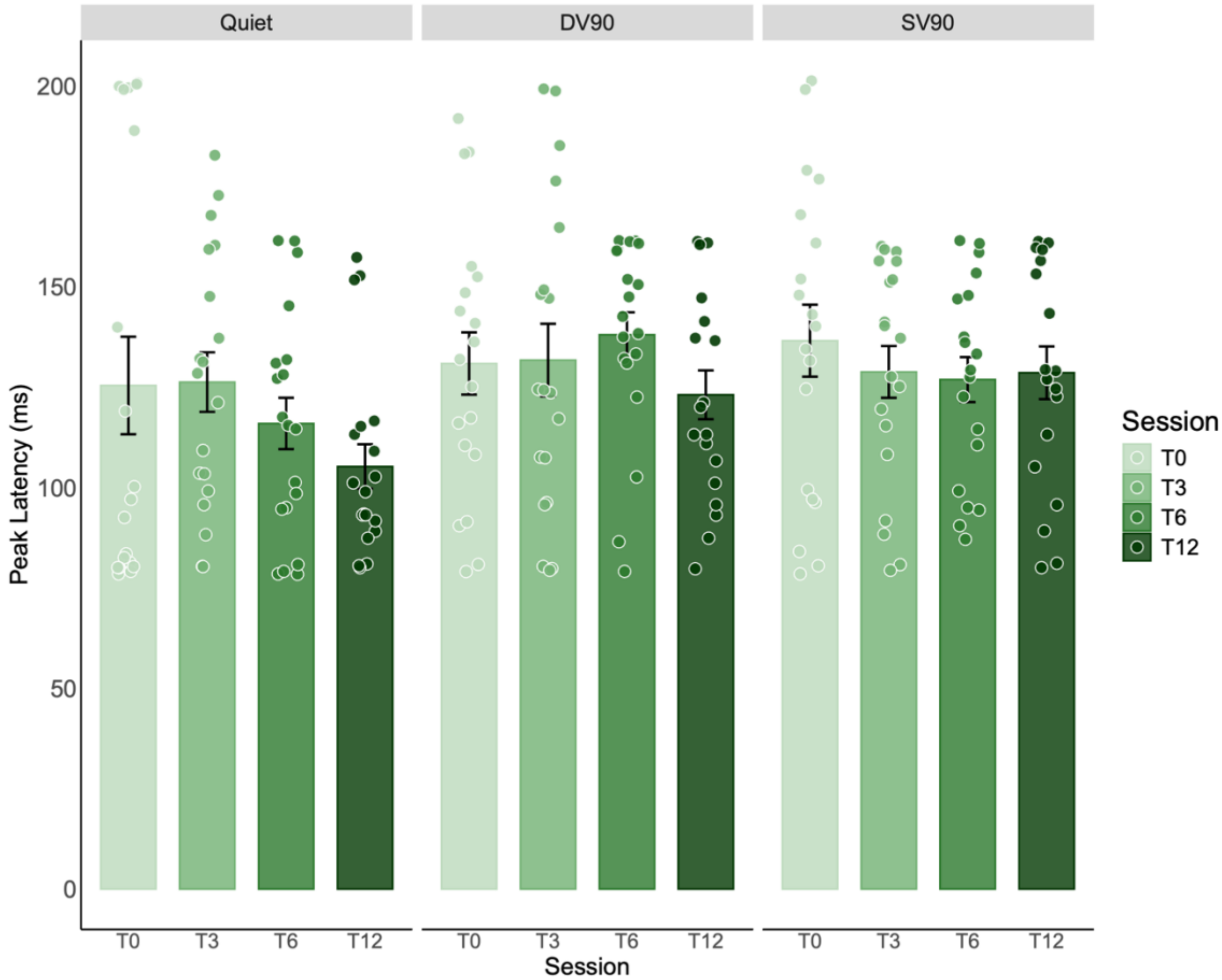

To determine whether the timing of the TRF response varied with listening context and post-implant experience, we examined TRF peak latency across conditions and sessions. Peak latency was defined as the local minimum of the TRF-N1 response averaged across three fronto-central electrodes within a 90–250 ms window. A repeated-measures ANOVA was then conducted with Condition and Session as within-subject factors.

There was a significant main effect of Condition, F(1.76, 31.60) = 5.01, *p* = .016, reflecting shorter latencies in Quiet (M = 118 ms, SE = 5.97) than in the spatial speech conditions DV90 (M = 131 ms, SE = 5.04) and SV90 (M = 130 ms, SE = 5.66). FDR-adjusted comparisons indicated that Quiet condition differed significantly from DV90 condition, *p* = .008, whereas the Quiet–SV90 comparison did not survive correction, *p* = .079, and DV90 did not differ from SV90, *p* = .986.

There was no significant main effect of Session, *F*(1.99, 35.78) = 1.91, *p* = .164, and no Condition × Session interaction, *F*(3.75, 67.43) = 1.18, *p* = .325. Thus, TRF-N1 peak latency varied by listening condition, with earlier latencies in Quiet than in DV90. However, there was no evidence that peak latency changed systematically across sessions or that the effect of condition differed over time.

Fig. S6. TRF-N1 Latency by Attention Streams. Error bars indicate the standard error of the mean.

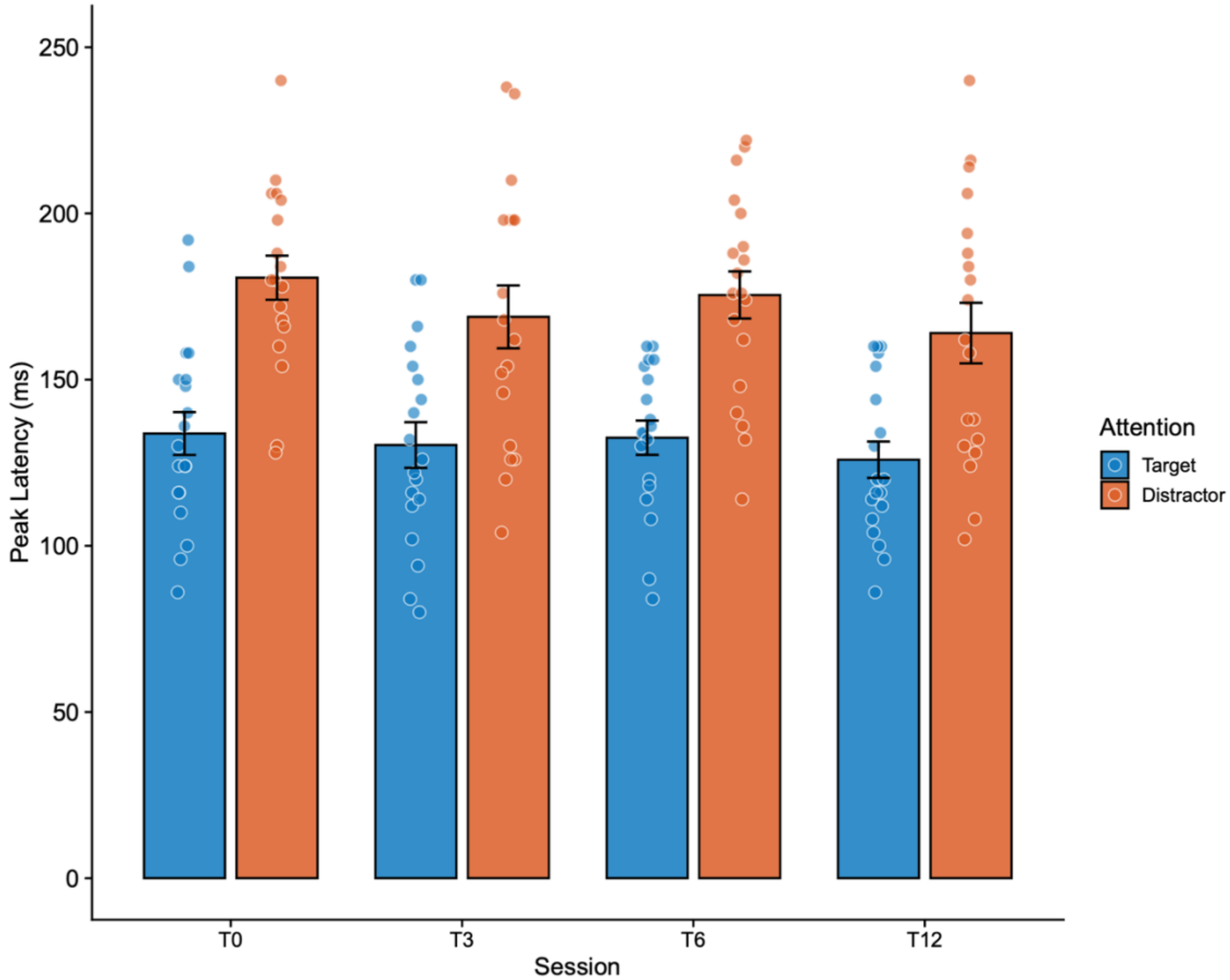

To determine whether TRF-N1 peak latency differed between task-relevant and task-irrelevant speech streams, we compared target- and distractor-related responses across sessions. A repeated-measures ANOVA was conducted with Attention and Session as within-subject factors.

There was a significant main effect of Attention, $F(1, 18) = 100.71$, $p < .001$. Target-related TRFs showed earlier peak latencies (M = 131 ms, SE = 4.85) than distractor-related TRFs (M = 175 ms, SE = 6.15).

There was no significant main effect of Session, $F(2.51, 45.20) = 1.54$, $p = .223$, and no significant Attention × Session interaction, $F(2.62, 47.21) = 0.75$, $p = .511$. Thus, target-related TRF-N1 responses peaked substantially earlier than distractor-related responses, but this latency difference did not change significantly across post-activation sessions.

Table S1. TRF prediction accuracy by condition and session. Prediction accuracy values were first averaged across EEG channels for each participant, for each condition and session. The table reports the group mean and standard deviation (SD) of these participant-level averages.

**Condition**		**T0**	**T3**	**T6**	**T12**
**Quiet**		0.0450 (0.0368)	0.0463 (0.0204)	0.0575 (0.0334)	0.0504 (0.0311)
**DV90**					
	Target	0.0274 (0.0206)	0.0359 (0.0150)	0.0340 (0.0145)	0.0384 (0.0205)
	Distractor	0.0057 (0.0106)	0.0085 (0.0091)	0.0069 (0.0076)	0.0080 (0.0089)
**SV90**					
	Target	0.0214 (0.0198)	0.0428 (0.0245)	0.0406 (0.0174)	0.0400 (0.0226)
	Distractor	0.0057 (0.0087)	0.0101 (0.0135)	0.0092 (0.0086)	0.0078 (0.0097)

Table S2. Linear mixed-effects model examining the association between TRF-N1 amplitude and listening effort across sessions and listening conditions. The model predicted listening effort from TRF-N1 amplitude, session, and listening condition, with participant included as a random intercept. Reference levels were T0 for the session and Quiet for the condition.

**Predictor**	**Estimate (β)**	**SE**	**df**	***t***	***p***
Intercept	6.11	0.38	67.25	15.96	**< .001**
TRF-N1 amplitude	3.95	1.57	192.07	2.52	**0.012**
Session: T12 vs. T0	–0.58	0.37	215.98	–1.57	0.117
Session: T3 vs. T0	−1.52	0.34	207.15	−4.54	< .001
Session: T6 vs. T0	−1.10	0.36	214.76	−3.05	0.003
Condition: DV90 vs. Quiet	2.93	0.29	205.14	10.22	**< .001**
Condition: SV90 vs. Quiet	3.03	0.29	205.18	10.58	**< .001**

_
